# Pathobiologic Roles of Epstein–Barr Virus-Encoded MicroRNAs in Human Lymphomas

**DOI:** 10.3390/ijms19041168

**Published:** 2018-04-12

**Authors:** Mohsen Navari, Maryam Etebari, Mostafa Ibrahimi, Lorenzo Leoncini, Pier Paolo Piccaluga

**Affiliations:** 1 Research Center of Advanced Technologies in Medicine, Torbat Heydariyeh University of Medical Sciences, Torbat Heydariyeh 9516915169, Iran; mohsen.navari@gmail.com (M.N.); maryam.etebari@gmail.com (M.E.); 2 Department of Experimental, Diagnostic, and Experimental Medicine, Bologna University School of Medicine, 40126 Bologna, Italy; 3 Department of Clinical Biochemistry, Faculty of Medical Sciences, Tarbiat Modares University, P.O. Box 14115-111, Tehran, Iran; mustafa.ibrahimi@modares.ac.ir; 4 Section of Pathology, Department of Medical Biotechnology, University of Siena, 53100 Siena, Italy; lorenzo.leoncini@dbm.unisi.it; 5 Euro-Mediterranean Institute of Science and Technology (IEMEST), 90139 Palermo, Italy; 6 Department of Pathology, Jomo Kenyatta University of Agriculture and Technology, P.O. Box 62000-00200, Nairobi, Kenya

**Keywords:** Epstein–Barr Virus, microRNA, human lymphoma, BamHI-A rightward transcript, *BART*, Bam HI fragment H rightward open reading frame 1, *BHRF1*

## Abstract

Epstein–Barr virus (EBV) is a human γ-herpesvirus implicated in several human malignancies, including a wide range of lymphomas. Several molecules encoded by EBV in its latent state are believed to be related to EBV-induced lymphomagenesis, among which microRNAs—small RNAs with a posttranscriptional regulating role—are of great importance. The genome of EBV encodes 44 mature microRNAs belonging to two different classes, including BamHI-A rightward transcript (*BART*) and Bam HI fragment H rightward open reading frame 1 (*BHRF1*), with different expression levels in different EBV latency types. These microRNAs might contribute to the pathogenetic effects exerted by EBV through targeting self mRNAs and host mRNAs and interfering with several important cellular mechanisms such as immunosurveillance, cell proliferation, and apoptosis. In addition, EBV microRNAs can regulate the surrounding microenvironment of the infected cells through exosomal transportation. Moreover, these small molecules could be potentially used as molecular markers. In this review, we try to present an updated and extensive view of the role of EBV-encoded miRNAs in human lymphomas.

## 1. Epstein–Barr Virus as an Oncovirus in Human Lymphomas

It is believed that viruses contribute to 15–20% of human malignancies [1]. Among the discovered oncoviruses to date, Epstein–Barr Virus (EBV), which is also named human herpesvirus 4 (HHV-4), is a key player in human malignancies [2]. It is a human γ-herpesvirus that is widely distributed in the human population, infecting more than 90–95% of its natural host for the entirety of its life [3]. As the first described human oncovirus, it was discovered in 1964 by Doctors. Anthony Epstein and Yvonne Barr (hence the name) in cultured cells isolated from endemic Burkitt lymphoma (BL) cases in sub-Saharan Africa [4,5]. Later, it became evident that an EBV infection has an oncogenic role not only in endemic BL (eBL), but also in several other cancers. Considering the scope of this paper, the described entities include eBL, sporadic BL (sBL), HIV-related BL (HIV-BL), Hodgkin lymphoma (HL), plasmablastic lymphoma (PBL), chronic lymphocytic leukemia (CLL), natural killer/T-cell lymphoma (NKTL), diffuse large B-cell lymphoma (DLBCL) of the elderly, HIV-related DLBCL (HIV-DLBCL), post-transplant lymphoproliferative disorder (PTLD), pyothorax-associated lymphoma (PAL), and methotrexate-associated lymphoproliferative disorder (MTX-LPD), among others [3,6,7,8,9,10,11].

### EBV Latency Programs

Like other members of the herpesvirus family, EBV displays different patterns of gene expression, which are known as latency programs [12,13]. In fact, the latency state(s) allow the virus to remain almost inactive in the host cells and maintain its genome with the minimum number of genes expressed, helping it, for example, to hide from the host’s immune system [14]. Upon the emergence of the appropriate conditions, the virus changes its state to the lytic phase, where it uses the host cell’s machinery for the production of viral progenies, and thus, infecting other cells horizontally [15,16]. More importantly, unlike α- and β-herpesviruses, which cause diseases in their lytic form, EBV, as a γ-herpesvirus, is known to induce most of its associated human diseases in the latent forms [17].

Based on the expression patterns of the main EBV latent proteins, several latency programs have been described for the virus (Table 1). These latent proteins include EBV-encoded nuclear antigen 1 (EBNA1), EBNA2, EBNA3s (3A, 3B and 3C), latent membrane protein1 (LMP1), and LMP-2s (A and B) [18,19]. Furthermore, EBV encodes two RNA molecules named Epstein–Barr virus (EBV)-encoded small RNAs (*EBER*s), which are used as targets for the detection of the virus in clinical samples by means of in situ hybridization (ISH) [20]. Regarding the topic of this review, i.e., the two families of microRNAs (miRNAs) encoded by EBV (BamHI-A rightward transcript or *BART*; BamHI fragment H rightward open reading frame 1 or *BHRF1*), there are some differences in their expression patterns between different latency types (Table 1). 

Along with the three main latency types of EBV, atypical instances have been reported too. For example, the BamHI W promoter (Wp)-restricted latency program was found in a subset of BLs, where a deletion of the *EBNA2* gene in the viral genome led to the expression of EBNA1 and EBNA3s, granting the infected cells an elevated resistance to apoptosis [21,22].

## 2. MicroRNAs

MicroRNAs are a class of small RNAs (almost 22 nucleotides in length) which were discovered in 1994 by Lee et al. in *C. elegans* [29]. They are used by cells to “fine-tune” gene expression at the post-transcriptional level, and they play a substantial role in different physiological processes such as differentiation, immune signaling, apoptosis, and proliferation [30,31]. 

miRNAs are transcribed as long pri-miRNA molecules (usually by RNA polymerase II), which are then cleaved by a complex containing RNase type III Drosha. The resulting molecules, pre-miRNAs, are almost 85 nucleotides in length and show a hairpin structure. After being transferred to the cytoplasm—a process mediated by exportin-5—the activity of another RNase (named Dicer) yields a miRNA/miRNA* duplex that is almost 20–22 nucleotides long. A mature miRNA (one of the two strands of the duplex) is then loaded onto an RNA-induced silencing complex (RISC) and guides it to the target site on the related mRNA molecules. In animals, the target sites are usually located on the 3′ UTR of mRNAs and exhibit full or partial complementary sequences to regulatory miRNAs. The final result is either the degradation of the target mRNA or the blockage of its translation, both of which lead to lowered expression levels of the related protein [32,33]. 

Being important regulatory molecules, the altered expression of miRNAs can play an important role in different human diseases such as cancers [33,34]. In addition, based on their target gene(s) and altered expression levels, these molecules are divided into two categories: onco- and tumor-suppressor miRNAs [33,35]. To date, numerous miRNAs with such characteristics have been described; however, the discovery of the fact that miRNAs are not solely produced by metazoans and plants, but also by viruses, has opened a new window in the field. The first ever reported viral miRNAs were described by Pfeffer and colleagues in EBV-infected B cells in 2004 [36].

## 3. EBV-Encoded MicroRNAs in Human Lymphomas

**Figure 1:**
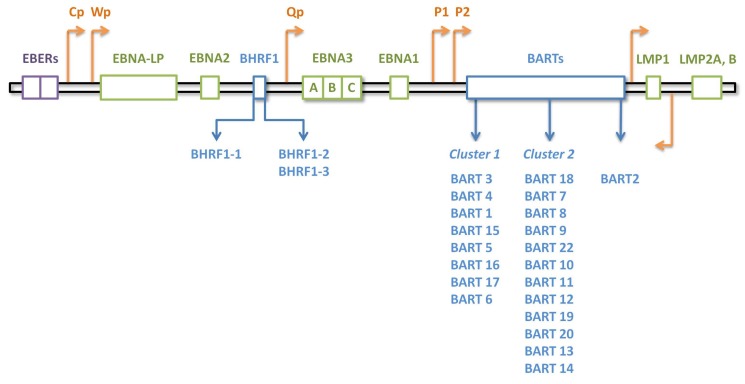
The structure of the EBV genome encoding latent products. Orange arrows indicate promoters. Blue boxes indicate miRNA coding regions. *BART*: BamHI-A rightward transcript; *BHRF1*: BamHI fragment H rightward open reading frame 1; Cp: C promoter; *EBERs*: EBV-encoded small RNAs; *EBNA1*: EBV-encoded nuclear antigen 1; *EBNA2*: EBV-encoded nuclear antigen 2; *EBNA3*: EBV-encoded nuclear antigen 3; *EBNA-LP*: EBV-encoded nuclear antigen leader protein; *LMP1*: latent membrane protein 1; *LMP2*: latent membrane protein 2; P1: promoter 1; P2: promoter 2; Qp: Q promoter; Wp: W promoter.

Among the human and animal viruses with their own miRNAs, herpesviruses are the main viruses. In the latest release of miRBase, 502 viral microRNAs have been annotated, 44 of which belong to EBV [37]. EBV microRNAs are transcribed from two regions: *BART*, comprising two clusters plus pre-miRNA for miR-BART2, and *BHRF1* cluster, encoding 40 and four mature miRNAs, respectively [38]. Figure 1 displays a schematic figure of the EBV genome encoding different latent products, including miRNAs, and the related promoters (Figure 1).

The transcription of the *BamHI* region produces large polyadenylated RNA molecules harboring several exons and introns, which are further alternatively spliced. The transcription is under the control of promoter 1 and 2 (P1 and P2), which are TATA-less promoters located approximately 400 nucleotides upstream of *BART* exon 1. Besides being subjected to reduced activity due to methylation, these promoters are regulated by several cellular transcription factors (further discussed below) [39]. Although the first four introns of the transcripts harbor the codons for the *BART* miRNAs, a study suggests that the processing of the pre-miRNAs occurs before the completion of the splicing reaction [40]. It is worth mentioning that for a long time, it was believed that these miRNAs were the only functional products of the transcription of the *BART* region [41]. Recent evidence, however, identifies functional long noncoding RNAs as other possible products [42].

On the other hand, the transcription of the *BHRF1* cluster of miRNAs is under the control of C and W promoters (Cp and Wp). The products of transcription are polycistronic transcripts which, apart from *BHRF1* miRNAs, encode the BHRF1 protein and some EBNA proteins as well [40,43]. 

### 3.1. EBV miRNA Expression Patterns

Several groups have applied high throughput technologies such as expression microarrays and next generation sequencing (NGS) methods in order to draw the expression patterns of EBV miRNAs in associated tumor samples, patient sera, and cell lines carrying different latency types. Some examples of the studied cancers include BL [6,9,38,44,45,46,47], HL [8,38], CLL [10], NKTL [11,48], PBL [6,38], DLBCL (of the elderly and HIV-related) [8], PTLD (systemic and central nervous system) [3,49], MTX-LPD [8], PAL [8], and lymphoblastoid cell lines (LCLs) [38,50,51]. 

Considering the heterogeneity observed in the reported data, creating a general consensus might seem overwhelming. The observed differences could be attributed to the usage of different technologies (e.g., microarray versus NGS), the sample type (cell line or tissue), or the sample quality (fresh versus formalin-fixed, paraffin-embedded) besides the number of the analyzed samples. Some patterns, however, are observable across these reports. 

It seems that the expression of *BART* miRNAs is more specific to the latency types I and II, with decreased or no expression in latency type III, in contrast to the expression of the *BHRF1* miRNA cluster in latency type III [3,8]. Meanwhile, *BHRF1* miRNAs can be detected in Wp-restricted latency too [52]. As mentioned before, different promoters are responsible for the transcription of *BART* or *BHRF1* miRNAs, which are regulated by several cancer- and immune system-related transcription factors. For example, c-MYC and CCAAT-enhancer-binding (C/EBP) proteins regulate P2 in a positive manner, while interferon regulatory factor (*IRF*) transcription factors IRF7 and IRF5 suppress P1-related expression [53]. On the other hand, NF-κB components positively regulate both promoters [54]. Hence the cellular context plays an important role in the regulation of the expression of these miRNAs which is mediated by both the viral latent proteins and/or (deregulated) cellular components, such as the NF-κB pathway, which is activated by LMP1 [54], and c-MYC, that is known to be constitutively expressed in BL [44]. It remains unclear, however, how the presence of LMP1 in latency type II and III does not lead to similar levels of *BART* miRNA expression. Although the reported methylation observed in EBV promoters could provide a possible hint for this phenomenon [55], more complex regulatory networks cannot be excluded.

Another notable observation in these reports is the various expression levels observed between the different members of the *BART* or *BHRF1* families in the same cell or tumor context. This difference could be as high as 50 times in the quantity of single miRNAs, despite each of the families being transcribed as single transcripts [40,43,52]. Several possibilities have been proposed, such as the effect of thermodynamics governing the secondary structure of pre-miRNA molecules on the efficiency of miRNA maturation [52,56], and a possible temporal sequence of cleavage of original transcripts [40]. Strikingly, recent data propose how different strains and types of EBV exhibit differences in their miRNA expression patterns, at least partially due to the presence of single nucleotide polymorphisms (SNPs) in their encoding DNA [57,58]. Thus, an interaction between different strains of the virus and the background of the infected cells most likely determines the expression level for each miRNA.

Conventional methods for the detection of EBV in patient samples mainly include immunohistochemistry (IHC), ISH, or serological methods such as anti-EBNA1 immunoglobulin G (IgG) detection [7,59]. These methods, however, have their intrinsic flaws, and in some cases they might be unsuccessful in identifying the target. For example, a very recent work published by us showed that while ISH and IHC failed to identify EBV presence in some BL cases (hence diagnosed as EBV-negative), Illumina high throughput sequencing recognized EBV genome presence and even some EBV miRNA expression in those cases [7]. More importantly, our results provided new and substantial support for the hit-and-run hypothesis in BL, which suggests that eliciting a heritable change in the gene expression pattern of the host cell may be followed by a complete loss of the genome of tumor viruses [7]. Similarly, other researchers reported detectable EBV miRNAs in CLL patients without an apparent history of an EBV infection [10]. These researchers, however, used anti-EBNA1 IgG detection as a means of revealing a previous EBV infection, which might not be the most appropriate method, since such a test would turn out negative for patients with viral chronic infection [60]. Alternatively, secondary anti-EBNA1 negativity in immunocompromised convalescent individuals such as tumor patients could provide another possible explanation for these researchers’ reported results [61].

### 3.2. Pathologic and Biologic Role of EBV miRNAs

In 90–100 million years of evolution, EBV has acquired the tools to survive in host cells and spread to other individuals [62]. It is thus perceivable that the miRNAs must have fundamental advantages for the virus since they have been retained in the viral genome [63]. Based on this observation, it is possible that the oncogenic roles attributed to EBV miRNAs could only be secondary effects of the mechanisms primarily developed for the long-term persistence of EBV in the host. 

One notable study supporting the essentiality of EBV miRNAs in its biology was recently published, where the authors found rarely occurring mutations in the *BART*- and *BHRF1*-encoding regions in EBV-associated lymphomas [64]. The viruses replicate and evolve much faster than their hosts; thus, this information suggests that EBV miRNAs are evolutionarily conserved genes.

According to the reported results, it is believed that EBV-encoded miRNAs play key roles in the pathobiology of the EBV life cycle and its associated cancers [39]. By targeting both cellular and viral mRNAs, they can modulate the latency/lytic phases of the EBV life cycle and also interfere with key cellular pathways engaged in fundamental cancer-related processes such as immunosurveillance, apoptosis, proliferation, cell cycle progression, transforming capacity, and other tumor-related mechanisms (discussed below). 

#### 3.2.1. EBV miRNAs Contribute to Evasion from Immunosurveillance

Being able to hide from the host immune system is an indispensable prerequisite for the latent virus. Some EBV proteins have immunoevasin characteristics; in other words, they have the potential to repel both the innate and the adaptive immune systems [65]. For instance, the most widely expressed viral protein, EBNA1, which is necessary for maintaining the viral genome inside the nucleolus, is protected from major histocompatibility complex (*MHC*) class I antigen presentation and the recognition of the infected cell by cytotoxic T cells—a property attributed to the Gly–Ala repeat (GAr) in its primary structure [66]. By targeting both the cellular components and the self-components, the miRNAs could provide the virus with further means to evade the immune system. This would potentially be more important in latency types I and II, since the latency type III is primarily observed in immunosuppressed or immunocompromised patients [3]. 

The reported cellular targets constitute important molecules that are engaged in both the innate and the adaptive immune system. This ensures the sustainability of the virus in all phases of the infection from its entrance into the host cell to lytic replication, latency, and lytic reactivation. Pattern recognition receptors are important components of innate immunity during the early phases of the infection and are responsible for detecting molecular patterns expressed by the invading pathogens [67]. Retinoic acid-inducible protein 1 (*RIG-I*) is a member of this superfamily which functions as a detector of viral RNA and is targeted by miR-BART6-3p [68]. Similarly, miR-BART15 interacts with the mRNA of another member named NLR family pyrin domain containing 3 (*NLRP3*). This affects the associated inflammasome complex, which is involved in the control of viral infections and normally plays a role in stimulating an immune response through the production of interleukin-1β (IL-1β) and interleukin-18 (IL-18) [69,70]. 

There are other molecules related to the innate immunity that are manipulated by EBV miRNAs. The interleukin 6 (*IL-6*) receptor B (IL-6RB), as demonstrated by us and others, is targeted by miR-BART6-3p [45,71]. Concordantly, the importin 7 (*IPO7*) inhibition by miR-BART16 and miR-BART3 could reduce the production of IL-6, which stresses the importance of the IL-6-related pathway in an EBV infection [72,73,74]. In a similar manner, the interleukin 1 (*IL-1*) receptor 1 is suppressed by miR-BHRF1-2-5p [75], and CREB-binding protein (*CREBBP*), a key transcriptional coactivator in type I Interferon (IFN) signaling, is a direct target of miR-BART16 [76]. 

Interferon regulatory factors are a family of transcription factors which are important players in the innate immune response. More importantly, as mentioned above, IRF7 and IRF5, which are induced by type I IFNs, can negatively regulate the expression of *BART* miRNAs through the P1 promoter, with the former suppressing Q promoter (Qp) -related transcription (connected to EBNA1 expression) as well [53]. Thus, by targeting these pathways, the *BART* miRNA(s) impair the innate immunity and also possibly guarantee their own and (some) latent protein expression.

The expression of viral proteins, at least during latency type II (considering EBNA1 as the sole EBV protein expressed in latency type I and immunosuppressed or immunocompromised status of patients expressing latency type III) and the lytic phase should normally trigger the cellular and humoral responses of the adaptive immune system. The signaling pathways related to the responses by the two main cell types of adaptive immunity (CD4 and CD8) are targeted by several EBV miRNAs of both families. The reported targets for the T helper cell response include interleukin-12 subunit beta (*IL-12B*) and lysosomal proteases such as legumain (*LGMN*), cathepsin B (*CTSB*), and interferon γ inducible protein 30 (*IFI30*). In addition, the suppression of a peptide transporter subunit named transporter associated with antigen processing 2 (*TAP2*), as well as the subsequent downregulation of another subunit, *TAP1*, provide evidence for the manipulation of the cytotoxic T cell response [77,78].

Besides influencing the host macromolecules, microRNAs are employed by EBV to control self-gene expression. In this way, the virus can regulate the latency or latency/lytic phase transition and limit the presentation of its antigens to the host immune system. Feederle et al. observed a higher expression of EBV latent proteins in cells infected with a mutant virus lacking *BHRF1* miRNAs, indicating a potential role of this cluster of microRNAs in the decrease of the antigenic load of virus-infected cells and in the regulation of viral latency [79]. Very interestingly, although it is capable of hiding from cytotoxic T cells, *EBNA1* is also targeted by EBV miRNAs, probably to limit its targeting by effector T cells [77]. Furthermore, the downregulation of the viral DNA polymerase, *BALF5*, by miR-BART2 is suggested to regulate lytic phase activation [80]. A paper by Sesto and colleagues, however, contradicts the latter findings, as the levels of the viral protein, *BZLF1*, that is necessary for lytic activation remained unchanged in the cells infected with mutant viruses lacking *BHRF1*, *BART*, or both miRNA families [81]. Further investigations are necessary to clarify this matter. 

Overall, this information highly supports that EBV miRNAs manipulate the immune response in different ways. In most cases, this simply leads to the impairment of antiviral responses with a nonlethal outcome, but it represents a major concern when the presence of EBV contributes to development of cancer. Above all, considering the importance of the cellular response of CD8 cells in combating cancer cells, EBV might be a major contributor to cancer progression.

#### 3.2.2. EBV miRNAs Interfere with Several Other Cancer-Related Mechanisms

The inhibition of apoptosis is one of the main processes employed by the virus that leads to its pathogenesis because the virus needs the host cell to survive and also needs to avoid transmitting potential danger signals that are released upon apoptosis such as viral particles, which could make the virus “more visible” to the human immune system [82,83]. Caspase 3 (*CASP3*), the main regulator of the execution of apoptosis, is targeted by several viral miRNAs consisting of miR-BART1-3p, miR-BART1-5p, miR-BART2-5p, miR-BART3-3p, miR-BART4-5p, miR-BART7-3p, miR-BART8-5p, miR-BART13-3p, miR-BART-16, and miR-BART22 [72,84]. There is most likely no other gene that has been reported to be targeted by so many viral miRNAs, which suggests the importance of this pathway for the virus. Furthermore, the translocase of outer mitochondrial membrane 22 (*TOMM22*) mRNA interacts with miR-BART16, resulting in the inhibition of the association between the pro-apoptotic protein BCL2 associated X (BAX) and the mitochondria, with the final outcome of BAX-induced apoptosis prevention [74]. Another example is the inhibition of PR/SET domain 1 (*PRDM1*) expression by miR-BHRF1-2 in LCLs [85]. In line with the latter information, EBNA1-induced hsa-miR-127 has previously been reported to downregulate *PRDM1* [32].

As well as avoiding apoptosis, the virus is also able to utilize its miRNAs to improve host cell proliferation, sometimes targeting important tumor suppressors. This can potentially increase the anti-apoptotic effect. For instance, phosphatase and tensin homolog (*PTEN*), a well-characterized tumor-suppressor gene which acts as a master regulator of cell proliferation, is a target of miR-BART6-3p, as well as the *BHRF1* cluster, while the latter can suppress the other known tumor suppressor, *p27* (protein 27, also known as *CDKN1B*) [45,86,87]. Another example is sphingosin-1-phosphate receptor 1 (*S1PR1*) which is downregulated by miR-BART16. In addition, the latter improves cell mobility as well—an important prerequisite for metastasis [48]. 

#### 3.2.3. Molecular Networks and Circuits Define More Complex Roles for EBV-Encoded MicroRNAs

Although the identification of single viral miRNA targets is a key step in understanding their pathologic role, it is known that one single mRNA could be targeted by several miRNAs, which suggests complex miRNA–miRNA networks, the effect of which could be additive/synergic [88,89]. Viral miRNAs are no exception, and very interestingly, this phenomenon could be extended to cellular transcripts cotargeted by viral and cellular deregulated miRNAs in the presence of the virus [90]. 

A paper by our group indicated that several host mRNAs which were differentially expressed between EBV-positive or EBV-negative HIV-BL could potentially be targeted by both viral and deregulated cellular miRNAs. We showed that the combined expression levels of the targets of both viral and cellular miRNAs could discriminate the two tumor categories better than each one alone, as indicated by gene expression profiling patterns [9]. In accordance with these results, a study conducted on Jijoye (Latency III) EBV-transformed B cells using high-throughput sequencing and crosslinking immunoprecipitation (HITS-CLIP) identified 90% of the targets of the 12 most copious EBV miRNAs to be also targeted by human miRNAs, though this occurred via distinct binding sites [91]. The synergism of hsa-miR-197 and miR-BART6-3p in targeting the *IL-6RB* and, similarly, hsa-miR-142 and miR-BART6-3p in the downregulation of *PTEN* provide us with more specific examples [71,86]. This can be extended even further, as in some cases, an EBV miRNA could interact with an mRNA at the same binding site of a cellular miRNA, e.g., targeting the *NLRP3* by hsa-miR-223 and EBV miR-BART15 at a shared 3′ UTR site [70].

MicroRNAs can also cooperate in the regulation of signaling circuits and networks by targeting different molecules related to them. A report described a circuit regulated by cellular and viral miRNAs in nasal natural killer cell lymphoma, which consisted of T-box Protein Expressed in T cells (*T-betPTEN*, *AKT*, and Rapamycin-Insensitive Companion of Mammalian Target of Rapamycin (*RICTOR*), and was regulated by miR-BART20-5p, hsa-miR-494-3p, and hsa-miR-142-3p [92]. This is a very good example of circuitous architecture of molecular interactions [89]. More extensively, Callegari and colleagues found more than 500 molecules of small ubiquitin-like modifier (*SUMO*) interactome to be potentially suppressed by EBV miRNAs [93]. This is a very interesting example, since important processes such as chromatin organization, stress, DNA damage, immune responses, and apoptosis are correlated to it. Interestingly, the extent of these networks and circuits is not limited to miRNAs and might include other noncoding RNAs as well, such as EBERs and miR-BART16, which are reported to corepress sphingosin-1-phosphate receptor 1 (*S1PR1*)—a process which contributes to NKTL tumor formation [48]. 

Furthermore, some researchers have tried to evaluate the function of EBV miRNA families from a more systematic point of view, evaluating the two miRNA families using mutant viruses that lacked either or both of them. In this regard, Vereide and colleagues found out that in the absence of any EBV-encoded product other than *BART* miRNAs, BL cells showed decreased apoptosis levels in comparison to non-*BART*-complemented cells. Furthermore, an increased proliferation mediated by *BART* miRNAs was observed [72]. Other researchers used a mutant EBV strain that lacked all three *BHRF1* miRNAs to infect B cells in vitro. Very interestingly, they found a 20-fold decrease in the B-cell transforming capacity of the mutant virus, slower growth, and a two-fold reduction in the entrance to the S phase of the cell cycle, as compared to the wild type virus [79]. Concordantly, the *BHRF1* cluster was proposed to play an important role in the promotion of cell cycle progression and the prevention of apoptosis [81]. It seems that such an effect is exerted by the *BHRF1* miRNA cluster targeting *PTEN* mRNA [87]. It is possible that studying each of these miRNAs separately, without the presence of other ones, would have resulted in different results, reflecting the importance of a systematic study of microRNAs.

With the advent of humanized mouse models for EBV infections, the systematic study of the EBV miRNAs has entered a new era, and it is hoped that light will be shed upon the matter, as in vivo systems provide researchers with considerable advantages. Upon infecting humanized mice with wild-type or mutant virus lacking the *BHRF1* miRNA cluster, Wahl et al. found that although *BHRF1* miRNAs do not enhance the overall oncogenic potential of EBV in vivo, they do facilitate the development of an acute systemic EBV infection [94]. This is somewhat in contrast to the reports described in the previous paragraph. Thus, further studies are necessary to confirm this data.

Considering the increasing number of discovered noncoding RNAs, such as long noncoding and circular RNAs, as well as newly identified miRNAs, the molecular networks describing their interactions should be the upcoming trend of noncoding RNA research, achieved above all by means of tools and techniques such as mathematical models and computer simulation. 

#### 3.2.4. The Pathogenetic Effect of Viral miRNAs Could Be Observed at Global Gene Expression Profile Level

The miRNA profiling data that was obtained using NGS technologies indicates that EBV-encoded miRNAs constitute a high percentage of the total miRNAs in the cells. This percentage could be as high as 34% [8]. Given that a single miRNA could target several mRNA molecules (primary targets) and the potential downstream genes affected by these deregulated targets (secondary targets) [32], the pathogenetic effect of these molecules should be observable in a global gene expression profile of the infected cells. In order to demonstrate such an effect, we have tried to use the targets of miR-BART6-3p (as a proof of principle) in some EBV-associated cancers. These targets were identified using transfection experiments of the miRNA or its inhibitor, followed by an assessment using gene expression profiling microarrays [3,44]. In this regard, we evaluated the expression of these targets in the context of EBV-positive versus EBV-negative BL gene expression profiles, and found them to be enriched in EBV-negative BL cases, which was in line with miR-BART6-3p absence of expression in this tumor [44]. In similar experiments where we compared EBV-positive BL (latency type I) versus EBV-positive PTLD (latency type III), these targets were upregulated in EBV-positive PTLD, where the expression of this miRNA was suppressed [3]. Considering these results, the role of *BART* miRNAs seemed to be more substantial in BL, which was in accordance with their expression levels, since, as opposed to PTLD, the immunocompetent state of a BL patient would not allow EBV to express a full array of its latent proteins. In PTLD, however, the viral protein might somehow surrogate the effect of the suppressed miRNAs [3]. 

#### 3.2.5. Exosomal Shuttle MicroRNAs Help EBV to Manipulate Its Surrounding Microenvironment

One of the means used for communication by adjacent cells in many eukaryotes, including humans, are exosomes—small (40–100 nm) vesicles that are secreted by parental cells (especially tumor cells) and are fused to the membrane of target cells. In doing so, exosomes transport a wide range of biological molecules, including proteins and nucleic acids, which could be delivered in their functional status [95]. In this light, exosomal microRNAs (also called exosomal shuttle microRNAs), which are available in different body fluids including serum, have recently become an important trend in tumor biology, and a considerable body of published literature deals with their role in carcinogenesis [96,97].

A primary study showed the transfer of some viral and cellular miRNAs from B cells to T cells after 1.5 h in a co-cultured system, giving the possibility of direct contact between the cells [98]. Later, however, it became evident that such a transfer does not necessarily need a direct contact, since primary immature monocyte-derived dendritic cells could be labeled using exosomes released from co-cultured LCL that were not in direct contact with them [99]. It is believed that these exosomes are taken up via caveola-dependent endocytosis [100].

Theoretically, the described effects of EBV miRNAs could be extended to the neighboring cells, giving the virus the potential to manipulate the tumor microenvironment for its benefit [101]. The targeting of the *NLRP3*-related inflammasome by miR-BART15 was also reported to happen in uninfected cells exposed to exosomes of virus-infected cells, leading to a weakened inflammatory response in neighboring uninfected cells [70]. In addition, Yogev et al. recently described a reduction in mitochondrial respiration characterized by a 25% decrease in oxygen consumption by fibroblasts exposed to such exosomes. This is a widespread cancer-related phenomenon known as the Warburg effect, which was followed by an increase in vascular endothelial growth factor A (*VEGFA*) expression [101]. It is possible, however, that the observed effect was related to other biomolecules transferred by exosomes.

More importantly, recent data have suggested the possibility of a specific viral miRNA packaging process in exosomes. In viral miRNA profiling experiments performed on LCLs and their exosomes, it was found that two viral miRNAs, i.e., miR-BART3 and miR-BHRF1-1, were more abundant in exosomes [50]. Another study, by contrast, reported that some viral miRNAs were enriched in LCLs, with none more expressed in exosomes, which suggests a tendency for these molecules to remain cell-associated [102]. 

In any case, there seems to be several mechanisms which can selectively accumulate specific miRNAs in exosomes. These exosomes can contain, apart from miRNAs, other EBV products such as latent proteins. Hence, this mechanism can be exploited by the virus to somehow dictate its signature to the surrounding non-infected cells, which also consist of cells that normally do not host it. To date, most of the conducted research has been focused on main hosts of EBV, i.e., B cells and fibroblasts. Studying other cells constituting tumor microenvironments can possibly help to further elucidate the tumorigenesis described for the virus. 

### 3.3. EBV miRNAs as Molecular Markers for Classification, Diagnosis, and Prognosis

Human miRNAs have been extensively used as diagnostic and prognostic molecular markers in different human cancers because of their abundance and stability [103,104]; thus, similar research has been applied to EBV miRNAs. For instance, high levels of several EBV miRNAs, including miR-BART2-5p, miR-BART7-3p, miR-BART13-3p, and miR-BART1-5p in the sera of patients with nasal NKTL turned out to be valuable for discriminating patients from healthy controls [11]. Similarly, the survival in CLL patients was found to be correlated with the expression of BHRF1-1 [10]. Concerning BL, we showed that based on EBV-encoded miRNA expression, it was possible to easily discriminate between EBV-positive and EBV-negative cases [9,44,45]; however, although EBV may certainly have a pathogenetic role in the first group, the clinical/epidemiological classification (endemic versus sporadic versus immunodeficiency-associated) still retains a substantial value, allowing for a broader view of these tumors. 

## 4. Conclusion Remarks

In this paper we evaluated the role of EBV-encoded miRNAs in B cell lymphomas and provided the readers with a brief description of the recent studies which indicate the importance of such a function. A summary of the discussed data is presented in Table 2.

With the advent of animal models for EBV infections, we expect more accurate and cancer-applicable results to come out. Furthermore, recent data obtained from NGS experiments of EBV strains could provide us with new insights relating to their different oncologic roles [57,105]. On the other hand, mechanisms exploited by other miRNA-encoding herpesviruses such as Kaposi sarcoma-associated herpesvirus and murine γ-herpesvirus 68 could guide researchers towards clues extendible to EBV [106].

It must be noted, however, that several other tumors such gastric carcinoma, nasopharyngeal carcinoma, breast cancer, and leiomyosarcomas might be correlated with EBV [107,108,109,110,111,112,113,114], further highlighting the importance of this virus in carcinogenesis. In addition, an increasing number of publications suggest that EBV-miRNA-related pathogenesis might not be limited to cancers or other known conditions such as infectious mononucleosis, since autoimmune diseases like multiple sclerosis and primary Sjögren’s syndrome have also been connected to EBV infection [115,116,117]. Altogether, these data point out the importance of the development of anti-EBV vaccines (reviewed in [118]). We believe that the development of such vaccines is not a choice, but is a necessity, and thus, we encourage more research on the issue. Similarly, the evidence that EBV-encoded miRNAs retain a pathogenetic role in human lymphomas may also represent the opportunity to target them with novel therapeutic agents, much like the approach taken with miR-BART7-3p and hsa-miR-155 [119,120].

## Figures and Tables

**Table 1:** Expression patterns of EBV-encoded products in different latency programs with references to selected cancers. Some entities may represent two types of latencies.

Latency Type	Expressed Product	Example	Reference
Type I	EBNA1, *EBERs*, *BART* miRNAs	BL, MTX-LPD, PBL	[9,23,24]
Type II	EBNA1, LMP1, LMP2s, *EBERs*, *BART* miRNAs	HL, PBL, MTX-LPD, NKTL, CLL, PTLD, DLBCL of the elderly	[6,23,24,25,26,27,28]
Type III	EBNA1, EBNA2, EBNA3s, LMP1, LMP2s, *EBERs*, *BART* miRNAs (non-to-low), *BHRF1*	PTLD, DLBCL of elderly, PAL, HIV-DLBCL, LCL	[3,8,28]

*BART*: BamHI-A rightward transcript; *BHRF1*: BamHI fragment H rightward open reading frame 1; BL: Burkitt lymphoma; CLL: chronic lymphocytic leukemia; DLBC: diffuse large B-cell lymphoma; *EBER*: EBV-encoded small RNA; EBNA: EBV-encoded nuclear antigen; HIV-DLBCL: HIV-related diffuse large B-cell lymphoma; HL: Hodgkin lymphoma; LCL: lymphoblastoid cell line; LMP: latent membrane protein; MTX-LPD: methotrexate-associated lymphoproliferative disorder; NKTL: natural killer/T-cell lymphoma; PAL: pyothorax-associated lymphoma; PBL: plasmablastic lymphoma; PTLD: post-transplant lymphoproliferative disorder.

**Table 2:** A summary of the discussed EBV miRNAs and their targets.

MicroRNA	Target	Related Process/Application	Reference
*BART* and *BHRF1* Families	*EBNA1*	Latency Regulation	[77]
*BHRF1* Family	*PTEN*	Cell Proliferation, Apoptosis	[87]
	*p27*	Cell Cycle Progression	[87]
	Unidentified	Transformation Capacity, Cell Cycle Progression	[79]
	Unidentified	Acute Systemic EBV Infection	[94]
	Latent Proteins	Latency Regulation	[79]
miR-BHRF1-1	Not Applicable	Survival Marker in CLL	[10]
miR-BHRF1-2	*CTSB*	CD4^+^ T Cell Response	[77,78]
	*PRDM1*	Cell Cycle Progression, Apoptosis	[85]
	*IL-12B*	CD4^+^ T Cell Response	[77,78]
miR-BHRF1-2-5p	IL-1 receptor	Innate Immunity	[75]
miR-BHRF1-3	*TAP2*	CD8^+^ T Cell Response	[77,78]
miR-BART1-3p	*IL-12B*	CD4^+^ T Cell Response	[77,78]
	*IFI30*	CD4^+^ T Cell Response	[77,78]
	*CASP3*	Apoptosis	[72]
miR-BART1-5p	*IFI30*	CD4^+^ T-Cell Response	[77,78]
	*CASP3*	Apoptosis	[84]
	Not Applicable	Diagnostic Marker for NKTL	[11]
miR-BART2-5p	*CTSB*	CD4^+^ T-Cell Response	[77,78]
	*LGMN*	CD4^+^ T-Cell Response	[77,78]
	*IL-12B*	CD4^+^ T-Cell Response	[77,78]
	*CASP3*	Apoptosis	[84]
	*BALF5*	Latency Regulation	[80]
	Not Applicable	Diagnostic Marker for NKTL	
miR-BART3-3p	*IPO7*	Innate Immunity	[72,74]
	*CASP3*	Apoptosis	[84]
miR-BART4-5p	*CASP3*	Apoptosis	[84]
miR-BART6-3p	*PTEN* *	Cell Proliferation, Apoptosis	[86]
	*IL-6RB* **	Innate Immunity	[71]
	*RIG-I*	Innate Immunity	[68]
miR-BART7-3p	*CASP3*	Apoptosis	[84]
	Not Applicable	Diagnostic Marker for NKTL	[11]
miR-BART8-5p	*CASP3*	Apoptosis	[84]
miR-BART10-3p	*IL-12B*	CD4^+^ T Cell Response	[77,78]
miR-BART13-3p	*CASP3*	Apoptosis	[84]
	Not Applicable	Diagnostic Marker for NKTL	[11]
miR-BART15	*NLRP3*	Innate Immunity	[70]
miR-BART16	*S1PR1*	Cell Growth/Mobility ***	[48]
	*CREBBP*	Innate Immunity	[76]
	*IPO7*	Innate Immunity	[72]
	*CASP3*	Apoptosis	[72]
	*TOMM22*	Apoptosis	[74]
miR-BART17	*TAP2*	CD8^+^ T-Cell Response	[77]
miR-BART20-5p	*T-bet* ****	Transcription Regulation of Cytotoxic NK Cells	[92]
miR-BART22	*CASP3*	Apoptosis	[84]
	*IL-12B*	CD4^+^ T Cell Response	[77,78]

* Synergism with has-miR-142; ** Synergism with has-miR-197; *** EBERs and miR-BART16 corepress the target; **** Along with hsa-miR-494-3p and hsa-miR-142-3p regulates PTEN–AKT–mTOR/RICTOR circuit.

## Abbreviations

*BART*	BamHI-A rightward transcript
*BAX*	BCL2 Associated X
*BHRF1*	BamHI fragment H rightward open reading frame 1
BL	Burkitt Lymphoma
*CASP3*	Caspase 3
*CDKN1B*	Cyclin Dependent Kinase Inhibitor 1b
*C/EBP*	CCAAT-Enhancer-Binding Protein
CLL	Chronic Lymphocytic Leukemia
*CREBBP*	CREB-Binding Protein
*CTSB*	cathepsin B
Cp	C Promoter
DLBCL	Diffuse Large B Cell Lymphoma
*EBER*s	EBV-Encoded small RNAs
eBL	endemic Burkitt Lymphoma
*EBNA*	EBV-Encoded Nuclear Antigen
*EBNA-LP*	EBV-Encoded Nuclear Antigen Leader Protein
EBV	Epstein–Barr Virus
HHV-4	Human Herpesvirus 4
HIV-BL	HIV-related Burkitt Lymphoma
HIV-DLBCL	HIV-related Diffuse Large B Cell Lymphoma
HL	Hodgkin Lymphoma
*IFI30*	Interferon Gamma Inducible Protein 30
*IFN*	Interferon
*IgG*	Immunoglobulin G
IHC	Immunohistochemistry
*IL-1*	Interleukin-1
*IL-1β*	Interleukin-1β
*IL-12B*	Interleukin-12 Subunit Beta
*IL-18*	Interleukin 18
*IL-6*	Interleukin-6
*IL-6RB*	Interleukin-6 Receptor B
*IPO7*	Importin 7
*IRF*	Interferon Regulatory Factor
ISH	In Situ Hybridization
LCL	Lymphoblastoid Cell Line
*LGMN*	Legumain
*LMP*	Latent Membrane Protein
*MHC*	Major Histocompatibility Complex
miRNA	microRNA
MTX-LPD	Methotrexate-Associated Lymphoproliferative Disorder
NKTL	Natural Killer/T Cell Lymphoma
*NLRP3*	NLR Family Pyrin Domain Containing 3
*p27*	protein 27
P1	Promoter 1
P2	Promoter 2
PAL	Pyothorax-Associated Lymphoma
PBL	Plasmablastic Lymphoma
*PRDM1*	pr/set domain 1
*PTEN*	Phosphatase and Tensin Homolog
PTLD	Post-Transplant Lymphoproliferative Disorder
Qp	Q Promoter
*RICTOR*	Rapamycin-Insensitive Companion of Mammalian Target of Rapamycin
*RIG-I*	Retinoic-Acid-Inducible Protein 1
*S1PR1*	Sphingosin-1-Phosphate Receptor 1
sBL	sporadic Burkitt Lymphoma
SNP	Single Nucleotide Polymorphism
*SUMO*	Small Ubiquitin-Like Modifier
*TAP*	Transporter Associated With Antigen Processing
*T-bet*	T-box Protein Expressed in T cells
*TOMM22*	Translocase of Outer Mitochondrial Membrane 22
*VEGFA*	Vascular Endothelial Growth Factor A
Wp	W Promoter

## References

[1] McLaughlin-Drubin M.E., Munger K. (2008). Viruses associated with human cancer. Biochim. Biophys. Acta.

[2] Esau D. (2017). Viral causes of lymphoma: The history of epstein–barr virus and human T-lymphotropic virus 1. Virology.

[3] Navari M., Fuligni F., Laginestra M.A., Etebari M., Ambrosio M.R., Sapienza M.R., Rossi M., de Falco G., Gibellini D., Tripodo C. (2014). Molecular signature of epstein–barr virus-positive burkitt lymphoma and post-transplant lymphoproliferative disorder suggest different roles for epstein–barr virus. Front. Microbiol..

[4] Epstein M., Achong B., Barr Y. (1964). Virus particles in cultured lymphoblasts from burkitt’s lymphoma. Lancet.

[5] Mesri E.A., Feitelson M.A., Munger K. (2014). Human viral oncogenesis: A cancer hallmarks analysis. Cell Host Microbe.

[6] Ambrosio M.R., Mundo L., Gazaneo S., Picciolini M., Vara P.S., Sayed S., Ginori A., lo Bello G., del Porro L., Navari M. (2017). MicroRNAs sequencing unveils distinct molecular subgroups of plasmablastic lymphoma. Oncotarget.

[7] Mundo L., Ambrosio M.R., Picciolini M., lo Bello G., Gazaneo S., del Porro L., Lazzi S., Navari M., Onyango N., Granai M. (2017). Unveiling another missing piece in EBV-driven lymphomagenesis: EBV-encoded micrornas expression in eber-negative burkitt lymphoma cases. Front. Microbiol..

[8] Sakamoto K., Sekizuka T., Uehara T., Hishima T., Mine S., Fukumoto H., Sato Y., Hasegawa H., Kuroda M., Katano H. (2017). Next-generation sequencing of mirnas in clinical samples of epstein–barr virus-associated B-cell lymphomas. Cancer Med..

[9] Navari M., Etebari M., de Falco G., Ambrosio M.R., Gibellini D., Leoncini L., Piccaluga P.P. (2015). The presence of epstein–barr virus significantly impacts the transcriptional profile in immunodeficiency-associated burkitt lymphoma. Front. Microbiol..

[10] Ferrajoli A., Ivan C., Ciccone M., Shimizu M., Kita Y., Ohtsuka M., D’Abundo L., Qiang J., Lerner S., Nouraee N. (2015). Epstein–barr virus microRNAs are expressed in patients with chronic lymphocytic leukemia and correlate with overall survival. eBioMedicine.

[11] Komabayashi Y., Kishibe K., Nagato T., Ueda S., Takahara M., Harabuchi Y. (2017). Circulating epstein–barr virus-encoded microRNAs as potential biomarkers for nasal natural killer/T-cell lymphoma. Hematol. Oncol..

[12] Grinde B. (2013). Herpesviruses: Latency and reactivation—Viral strategies and host response. J. Oral Microbiol..

[13] Speck S.H., Ganem D. (2010). Viral latency and its regulation: Lessons from the γ-herpesviruses. Cell Host Microbe.

[14] Munz C. (2004). Epstein–barr virus nuclear antigen 1: From immunologically invisible to a promising T cell target. J. Exp. Med..

[15] Chen H.S., Lu F., Lieberman P.M. (2013). Epigenetic regulation of EBV and KSHV latency. Curr. Opin. Virol..

[16] Schelcher C., Valencia S., Delecluse H.J., Hicks M., Sinclair A.J. (2005). Mutation of a single amino acid residue in the basic region of the epstein–barr virus (EBV) lytic cycle switch protein zta (bzlf1) prevents reactivation of ebv from latency. J. Virol..

[17] Kenney S.C. (2007). Reactivation and Lytic Replication of EBV.

[18] Grywalska E., Rolinski J. (2015). Epstein–barr virus-associated lymphomas. Semin. Oncol..

[19] Ok C.Y., Li L., Young K.H. (2015). EBV-driven B-cell lymphoproliferative disorders: From biology, classification and differential diagnosis to clinical management. Exp. Mol. Med..

[20] Weiss L.M., Chen Y.-Y. (2013). EBER in situ hybridization for epstein–barr virus. Hematological Malignancies.

[21] Kelly G.L., Stylianou J., Rasaiyaah J., Wei W., Thomas W., Croom-Carter D., Kohler C., Spang R., Woodman C., Kellam P. (2013). Different patterns of epstein–barr virus latency in endemic burkitt lymphoma (BL) lead to distinct variants within the BL-associated gene expression signature. J. Virol..

[22] Kelly G.L., Milner A.E., Tierney R.J., Croom-Carter D.S., Altmann M., Hammerschmidt W., Bell A.I., Rickinson A.B. (2005). Epstein–barr virus nuclear antigen 2 (EBNA2) gene deletion is consistently linked with EBNA3A,-3b, and-3c expression in burkitt’s lymphoma cells and with increased resistance to apoptosis. J. Virol..

[23] Gion Y., Iwaki N., Takata K., Takeuchi M., Nishida K., Orita Y., Tachibana T., Yoshino T., Sato Y. (2017). Clinicopathological analysis of methotrexate-associated lymphoproliferative disorders: Comparison of diffuse large B-cell lymphoma and classical hodgkin lymphoma types. Cancer Sci..

[24] Young L.S., Yap L.F., Murray P.G. (2016). Epstein–barr virus: More than 50 years old and still providing surprises. Nat. Rev. Cancer.

[25] Kis L.L., Gerasimčik N., Salamon D., Persson E.K., Nagy N., Klein G., Severinson E., Klein E. (2011). Stat6 signaling pathway activated by the cytokines IL-4 and IL-13 induces expression of the epstein–barr virus—Encoded protein LMP-1 in absence of EBNA-2: Implications for the type II EBV latent gene expression in hodgkin lymphoma. Blood.

[26] Chiang A.K., Tao Q., Srivastava G., Ho F. (1996). Nasal NK-and T-cell lymphomas share the same type of epstein-barr virus latency as nasopharyngeal carcinoma and hodgkin’s disease. Int. J. Cancer.

[27] Laytragoon-Lewin N., Chen F., Avila-Cariño J., Zou J.Z., Mellstedt H., Ernberg I., Klein G. (1995). Epstein–barr virus (EBV)-carrying cells of a chronic lymphocytic leukemia (CLL) subpopulation express EBNA1 and lmps but not EBNA2 in vivo. Int. J. Cancer.

[28] Castillo J.J., Beltran B.E., Miranda R.N., Paydas S., Winer E.S., Butera J.N. (2011). Epstein–barr virus–positive diffuse large B-cell lymphoma of the elderly: What we know so far. Oncologist.

[29] Lee R.C., Feinbaum R.L., Ambros V. (1993). The *C. elegans* heterochronic gene LIN-4 encodes small RNAs with antisense complementarity to LIN-14. Cell.

[30] Bhaskaran M., Mohan M. (2014). Micrornas: History, biogenesis, and their evolving role in animal development and disease. Vet. Pathol..

[31] Iorio M.V., Croce C.M. (2017). Microrna dysregulation in cancer: Diagnostics, monitoring and therapeutics. A comprehensive review. EMBO Mol. Med..

[32] Onnis A., Navari M., Antonicelli G., Morettini F., Mannucci S., de Falco G., Vigorito E., Leoncini L. (2012). Epstein–barr nuclear antigen 1 induces expression of the cellular microrna HSA-MIR-127 and impairing b-cell differentiation in EBV-infected memory B cells. New insights into the pathogenesis of burkitt lymphoma. Blood Cancer J..

[33] Peng Y., Croce C.M. (2016). The role of micrornas in human cancer. Signal Transduct. Target. Ther..

[34] Hayes J., Peruzzi P.P., Lawler S. (2014). MicroRNAs in cancer: Biomarkers, functions and therapy. Trends Mol. Med..

[35] Adams B.D., Anastasiadou E., Esteller M., He L., Slack F.J. (2015). The inescapable influence of noncoding RNAs in cancer. Cancer Res..

[36] Pfeffer S., Zavolan M., Grasser F.A., Chien M., Russo J.J., Ju J., John B., Enright A.J., Marks D., Sander C. (2004). Identification of virus-encoded microRNAs. Science.

[37] Kozomara A., Griffiths-Jones S. (2014). Mirbase: Annotating high confidence microRNAs using deep sequencing data. Nucleic Acids Res..

[38] Qiu J., Cosmopoulos K., Pegtel M., Hopmans E., Murray P., Middeldorp J., Shapiro M., Thorley-Lawson D.A. (2011). A novel persistence associated ebv mirna expression profile is disrupted in neoplasia. PLoS Pathog..

[39] Skalsky R.L., Cullen B.R. (2015). Ebv noncoding rnas. Epstein–Barr Virus.

[40] Edwards R.H., Marquitz A.R., Raab-Traub N. (2008). Epstein–barr virus bart microRNAs are produced from a large intron prior to splicing. J. Virol..

[41] Al-Mozaini M., Bodelon G., Karstegl C.E., Jin B., Al-Ahdal M., Farrell P.J. (2009). Epstein–barr virus bart gene expression. J. Gen. Virol..

[42] Marquitz A.R., Mathur A., Edwards R.H., Raab-Traub N. (2015). Host Gene Expression Is Regulated by Two Types of Noncoding RNAs Transcribed from the Epstein-Barr Virus BamHI A Rightward Transcript Region. J. Virol..

[43] Poling B.C., Price A.M., Luftig M.A., Cullen B.R. (2017). The epstein–barr virus MIR-BHRF1 microRNAs regulate viral gene expression in CIS. Virology.

[44] Piccaluga P.P., Navari M., de Falco G., Ambrosio M.R., Lazzi S., Fuligni F., Bellan C., Rossi M., Sapienza M.R., Laginestra M.A. (2016). Virus-encoded microrna contributes to the molecular profile of EBV-positive burkitt lymphomas. Oncotarget.

[45] Ambrosio M.R., Navari M., Di Lisio L., Leon E.A., Onnis A., Gazaneo S., Mundo L., Ulivieri C., Gomez G., Lazzi S. (2014). The epstein–barr-encoded BART-6-3p microrna affects regulation of cell growth and immuno response in burkitt lymphoma. Infect. Agent Cancer.

[46] Oduor C.I., Movassagh M., Kaymaz Y., Chelimo K., Otieno J., Ong’echa J.M., Moormann A.M., Bailey J.A. (2017). Human and epstein–barr virus mirna profiling as predictive biomarkers for endemic burkitt lymphoma. Front. Microbiol..

[47] Hooykaas M.J., Kruse E., Wiertz E.J., Lebbink R.J. (2016). Comprehensive profiling of functional epstein–barr virus mirna expression in human cell lines. BMC Genom..

[48] Alles J., Menegatti J., Motsch N., Hart M., Eichner N., Reinhardt R., Meister G., Grasser F.A. (2016). miRNA expression profiling of epstein–barr virus-associated NKTL cell lines by illumina deep sequencing. FEBS Open Bio.

[49] Fink S.E., Gandhi M.K., Nourse J.P., Keane C., Jones K., Crooks P., Johrens K., Korfel A., Schmidt H., Neumann S. (2014). A comprehensive analysis of the cellular and EBV-specific micrornaome in primary CNS PTLD identifies different patterns among EBV-associated tumors. Am. J. Transplant..

[50] Gallo A., Vella S., Miele M., Timoneri F., di Bella M., Bosi S., Sciveres M., Conaldi P.G. (2017). Global profiling of viral and cellular non-coding RNAs in epstein–barr virus-induced lymphoblastoid cell lines and released exosome cargos. Cancer Lett..

[51] Hoshina S., Sekizuka T., Kataoka M., Hasegawa H., Hamada H., Kuroda M., Katano H. (2016). Profile of exosomal and intracellular microrna in γ-herpesvirus-infected lymphoma cell lines. PLoS ONE.

[52] Amoroso R., Fitzsimmons L., Thomas W.A., Kelly G.L., Rowe M., Bell A.I. (2011). Quantitative studies of epstein–barr virus-encoded microRNAs provide novel insights into their regulation. J. Virol..

[53] Chen H., Huang J., Wu F.Y., Liao G., Hutt-Fletcher L., Hayward S.D. (2005). Regulation of expression of the epstein–barr virus bamhi-A rightward transcripts. J. Virol..

[54] Verhoeven R.J.A., Tong S., Zhang G., Zong J., Chen Y., Jin D.-Y., Chen M.-R., Pan J., Chen H. (2016). NF-κB signaling regulates expression of epstein–barr virus bart microRNAs and long noncoding RNAs in nasopharyngeal carcinoma. J. Virol..

[55] Kim D.N., Song Y.-J., Lee S.K. (2011). The role of promoter methylation in epstein–barr virus (EBV) microrna expression in EBV-infected B cell lines. Exp. Mol. Med..

[56] Haar J., Contrant M., Bernhardt K., Feederle R., Diederichs S., Pfeffer S., Delecluse H.-J. (2015). The expression of a viral microrna is regulated by clustering to allow optimal B cell transformation. Nucleic Acids Res..

[57] Tsai M.H., Lin X., Shumilov A., Bernhardt K., Feederle R., Poirey R., Kopp-Schneider A., Pereira B., Almeida R., Delecluse H.J. (2017). The biological properties of different epstein–barr virus strains explain their association with various types of cancers. Oncotarget.

[58] Correia S., Palser A., Elgueta Karstegl C., Middeldorp J.M., Ramayanti O., Cohen J.I., Hildesheim A., Fellner M.D., Wiels J., White R.E. (2017). Natural variation of epstein–barr virus genes, proteins, and primary microRNA. J. Virol..

[59] De Paschale M., Clerici P. (2012). Serological diagnosis of epstein–barr virus infection: Problems and solutions. World J. Virol..

[60] Miller G., Grogan E., Rowe D., Rooney C., Heston L., Eastman R., Andiman W., Niederman J., Lenoir G., Henle W. (1987). Selective lack of antibody to a component of EB nuclear antigen in patients with chronic active epstein–barr virus infection. J. Infect. Dis..

[61] Vetter V., Kreutzer L., Bauer G. (1994). Differentiation of primary from secondary anti-EBNA-1-negative cases by determination of avidity of VCA-IGG. Clin. Diagn. Virol..

[62] Hjalgrim H., Friborg J., Melbye M. (2007). The Epidemiology of EBV and Its Association with Malignant Disease.

[63] Martin J.N. (2007). The Epidemiology of Kshv and Its Association with Malignant Disease.

[64] Sunagawa K., Hishima T., Fukumoto H., Hasegawa H., Katano H. (2017). Conserved sequences of bart and bhrf regions encoding viral microRNAs in epstein–barr virus-associated lymphoma. BMC Res. Notes.

[65] Ressing M.E., van Gent M., Gram A.M., Hooykaas M.J., Piersma S.J., Wiertz E.J. (2015). Immune evasion by epstein–barr virus. Epstein–Barr Virus.

[66] Apcher S., Daskalogianni C., Manoury B., Fåhraeus R. (2010). Epstein–barr virus-encoded EBNA1 interference with MHC class I antigen presentation reveals a close correlation between mrna translation initiation and antigen presentation. PLoS Pathog..

[67] Thompson M.R., Kaminski J.J., Kurt-Jones E.A., Fitzgerald K.A. (2011). Pattern recognition receptors and the innate immune response to viral infection. Viruses.

[68] Lu Y., Qin Z., Wang J., Zheng X., Lu J., Zhang X., Wei L., Peng Q., Zheng Y., Ou C. (2017). Epstein–barr virus miR-BART6-3p inhibits the RIG-I pathway. J. Innate Immun..

[69] Chen I.Y., Ichinohe T. (2015). Response of host inflammasomes to viral infection. Trends Microbiol..

[70] Haneklaus M., Gerlic M., Kurowska-Stolarska M., Rainey A.A., Pich D., McInnes I.B., Hammerschmidt W., O’Neill L.A., Masters S.L. (2012). Cutting edge: MiR-223 and EBV miR-bart15 regulate the NLRP3 inflammasome and IL-1β production. J. Immunol..

[71] Zhang Y.M., Yu Y., Zhao H.P. (2017). EBVbart63p and cellular microRNA197 compromise the immune defense of host cells in ebvpositive burkitt lymphoma. Mol. Med. Rep..

[72] Vereide D.T., Seto E., Chiu Y.F., Hayes M., Tagawa T., Grundhoff A., Hammerschmidt W., Sugden B. (2014). Epstein–barr virus maintains lymphomas via its mirnas. Oncogene.

[73] Yang I.V., Wade C.M., Kang H.M., Alper S., Rutledge H., Lackford B., Eskin E., Daly M.J., Schwartz D.A. (2009). Identification of novel genes that mediate innate immunity using inbred mice. Genetics.

[74] Dolken L., Malterer G., Erhard F., Kothe S., Friedel C.C., Suffert G., Marcinowski L., Motsch N., Barth S., Beitzinger M. (2010). Systematic analysis of viral and cellular microrna targets in cells latently infected with human gamma-herpesviruses by risc immunoprecipitation assay. Cell Host Microbe.

[75] Skinner C.M., Ivanov N.S., Barr S.A., Chen Y., Skalsky R.L. (2017). An epstein–barr virus microRNA blocks interleukin-1 (IL-1) signaling by targeting IL-1 receptor 1. J. Virol..

[76] Hooykaas M.J.G., van Gent M., Soppe J.A., Kruse E., Boer I.G.J., van Leenen D., Groot Koerkamp M.J.A., Holstege F.C.P., Ressing M.E., Wiertz E. (2017). EBV microrna BART16 suppresses type I ifn signaling. J. Immunol..

[77] Albanese M., Tagawa T., Bouvet M., Maliqi L., Lutter D., Hoser J., Hastreiter M., Hayes M., Sugden B., Martin L. (2016). Epstein–barr virus microRNAs reduce immune surveillance by virus-specific CD8^+^ T cells. Proc. Natl. Acad. Sci. USA.

[78] Tagawa T., Albanese M., Bouvet M., Moosmann A., Mautner J., Heissmeyer V., Zielinski C., Lutter D., Hoser J., Hastreiter M. (2016). Epstein–barr viral mirnas inhibit antiviral CD4^+^ T cell responses targeting IL-12 and peptide processing. J. Exp. Med..

[79] Feederle R., Linnstaedt S.D., Bannert H., Lips H., Bencun M., Cullen B.R., Delecluse H.-J. (2011). A viral microrna cluster strongly potentiates the transforming properties of a human herpesvirus. PLoS Pathog..

[80] Barth S., Pfuhl T., Mamiani A., Ehses C., Roemer K., Kremmer E., Jäker C., Höck J., Meister G., Grässer F.A. (2007). Epstein–barr virus-encoded microrna miR-bart2 down-regulates the viral DNA polymerase BALF5. Nucleic Acids Res..

[81] Seto E., Moosmann A., Grömminger S., Walz N., Grundhoff A., Hammerschmidt W. (2010). MicroRNAs of epstein–barr virus promote cell cycle progression and prevent apoptosis of primary human B cells. PLoS Pathog..

[82] Ning S. (2011). Innate immune modulation in EBV infection. Herpesviridae.

[83] Everett H., McFadden G. (2001). Viruses and apoptosis: Meddling with mitochondria. Virology.

[84] Harold C., Cox D., Riley K.J. (2016). Epstein–barr viral microRNAs target caspase 3. Virol. J..

[85] Ma J., Nie K., Redmond D., Liu Y., Elemento O., Knowles D.M., Tam W. (2016). EBV-miR-BHRF1-2 targets PRDM1/BLIMP1: Potential role in EBV lymphomagenesis. Leukemia.

[86] Zhou L., Bu Y., Liang Y., Zhang F., Zhang H., Li S. (2016). Epstein–barr virus (EBV)-bamhi-a rightward transcript (bart)-6 and cellular microRNA-142 synergistically compromise immune defense of host cells in EBV-positive burkitt lymphoma. Med. Sci. Monit..

[87] Bernhardt K., Haar J., Tsai M.H., Poirey R., Feederle R., Delecluse H.J. (2016). A viral microRNA cluster regulates the expression of PTEN, p27 and of a BCL-2 homolog. PLoS Pathog..

[88] Zhang J., Duy Le T., Liu L., He J., Li J. (2016). Identifying mirna synergistic regulatory networks in heterogeneous human data via network motifs. Mol. Biosyst..

[89] Anastasiadou E., Jacob L.S., Slack F.J. (2018). Non-coding RNA networks in cancer. Nat. Rev. Cancer.

[90] Anastasiadou E., Garg N., Bigi R., Yadav S., Campese A.F., Lapenta C., Spada M., Cuomo L., Botta A., Belardelli F. (2015). Epstein–barr virus infection induces miR-21 in terminally differentiated malignant B cells. Int. J. Cancer.

[91] Riley K.J., Rabinowitz G.S., Yario T.A., Luna J.M., Darnell R.B., Steitz J.A. (2012). EBV and human microRNAs co-target oncogenic and apoptotic viral and human genes during latency. EMBO J..

[92] Chen H.H., Huang W.T., Yang L.W., Lin C.W. (2015). The PTEN-AKT-MTOR/RICTOR pathway in nasal natural killer cell lymphoma is activated by miR-494-3p via PTEN but inhibited by miR-142-3p via rictor. Am. J. Pathol..

[93] Callegari S., Gastaldello S., Faridani O.R., Masucci M.G. (2014). Epstein–barr virus encoded microRNAs target sumo-regulated cellular functions. FEBS J..

[94] Wahl A., Linnstaedt S.D., Esoda C., Krisko J.F., Martinez-Torres F., Delecluse H.J., Cullen B.R., Garcia J.V. (2013). A cluster of virus-encoded microRNAs accelerates acute systemic epstein–barr virus infection but does not significantly enhance virus-induced oncogenesis in vivo. J. Virol..

[95] Teow S.Y., Liew K., Khoo A.S., Peh S.C. (2017). Pathogenic role of exosomes in epstein–barr virus (EBV)-associated cancers. Int. J. Biol. Sci..

[96] Zhao L., Liu W., Xiao J., Cao B. (2015). The role of exosomes and “exosomal shuttle microrna” in tumorigenesis and drug resistance. Cancer Lett..

[97] Schwarzenbach H. (2017). Clinical relevance of circulating, cell-free and exosomal microRNAs in plasma and serum of breast cancer patients. Oncol. Res. Treat..

[98] Rechavi O., Erlich Y., Amram H., Flomenblit L., Karginov F.V., Goldstein I., Hannon G.J., Kloog Y. (2009). Cell contact-dependent acquisition of cellular and viral nonautonomously encoded small rnas. Genes Dev..

[99] Pegtel D.M., Cosmopoulos K., Thorley-Lawson D.A., van Eijndhoven M.A., Hopmans E.S., Lindenberg J.L., de Gruijl T.D., Wurdinger T., Middeldorp J.M. (2010). Functional delivery of viral miRNAs via exosomes. Proc. Natl. Acad. Sci. USA.

[100] Nanbo A., Kawanishi E., Yoshida R., Yoshiyama H. (2013). Exosomes derived from epstein–barr virus-infected cells are internalized via caveola-dependent endocytosis and promote phenotypic modulation in target cells. J. Virol..

[101] Yogev O., Henderson S., Hayes M.J., Marelli S.S., Ofir-Birin Y., Regev-Rudzki N., Herrero J., Enver T. (2017). Herpesviruses shape tumour microenvironment through exosomal transfer of viral microRNAs. PLoS Pathog..

[102] Koppers-Lalic D., Hackenberg M., Bijnsdorp I.V., van Eijndhoven M.A.J., Sadek P., Sie D., Zini N., Middeldorp J.M., Ylstra B., de Menezes R.X. (2014). Nontemplated nucleotide additions distinguish the small RNA composition in cells from exosomes. Cell Rep..

[103] Faruq O., Vecchione A. (2015). MicroRNA: Diagnostic perspective. Front. Med..

[104] De Souza M.F., Kuasne H., de Camargo Barros-Filho M., Cilião H.L., Marchi F.A., Fuganti P.E., Paschoal A.R., Rogatto S.R., de Syllos Cólus I.M. (2017). Circulating mrnas and miRNAs as candidate markers for the diagnosis and prognosis of prostate cancer. PLoS ONE.

[105] Palser A.L., Grayson N.E., White R.E., Corton C., Correia S., Ba Abdullah M.M., Watson S.J., Cotten M., Arrand J.R., Murray P.G. (2015). Genome diversity of epstein–barr virus from multiple tumor types and normal infection. J. Virol..

[106] Feldman E.R., Kara M., Coleman C.B., Grau K.R., Oko L.M., Krueger B.J., Renne R., van Dyk L.F., Tibbetts S.A. (2014). Virus-encoded microRNAs facilitate gammaherpesvirus latency and pathogenesis in vivo. MBio.

[107] Richardson A.K., Currie M.J., Robinson B.A., Morrin H., Phung Y., Pearson J.F., Anderson T.P., Potter J.D., Walker L.C. (2015). Cytomegalovirus and epstein–barr virus in breast cancer. PLoS ONE.

[108] Pai T., Gupta S., Gurav M., Nag S., Shet T., Patil A., Desai S. (2018). Evidence for the association of epstein–barr virus in breast cancer in indian patients using in-situ hybridization technique. Breast J..

[109] Miettinen M. (2014). Smooth muscle tumors of soft tissue and non-uterine viscera: Biology and prognosis. Mod. Pathol..

[110] Kang B.W., Choi Y., Kwon O.K., Lee S.S., Chung H.Y., Yu W., Bae H.I., Seo A.N., Kang H., Lee S.K. (2017). High level of viral microRNA-BART20-5p expression is associated with worse survival of patients with epstein–barr virus-associated gastric cancer. Oncotarget.

[111] Treece A.L., Duncan D.L., Tang W., Elmore S., Morgan D.R., Dominguez R.L., Speck O., Meyers M.O., Gulley M.L. (2016). Gastric adenocarcinoma microrna profiles in fixed tissue and in plasma reveal cancer-associated and epstein–barr virus-related expression patterns. Lab. Investig..

[112] Gao W., Li Z.H., Chen S., Chan J.Y., Yin M., Zhang M.J., Wong T.S. (2017). Epstein–barr virus encoded microRNA BART7 regulates radiation sensitivity of nasopharyngeal carcinoma. Oncotarget.

[113] Chan J.Y., Wong S.T., Wei W.I. (2015). The role of epstein–barr virus-encoded microRNA BART7 status of resection margins in the prediction of local recurrence after salvage nasopharyngectomy for recurrent nasopharyngeal carcinoma. Cancer.

[114] Hsu C.Y., Yi Y.H., Chang K.P., Chang Y.S., Chen S.J., Chen H.C. (2014). The epstein–barr virus-encoded microrna miR-BART9 promotes tumor metastasis by targeting E-cadherin in nasopharyngeal carcinoma. PLoS Pathog..

[115] Wang Y.F., He D.D., Liang H.W., Yang D., Yue H., Zhang X.M., Wang R., Li B., Yang H.X., Liu Y. (2017). The identification of up-regulated EBV-miR-BHRF1-2-5p targeting MALT1 and EBV-MIR-BHRF1-3 in the circulation of patients with multiple sclerosis. Clin. Exp. Immunol..

[116] Fernandez-Menendez S., Fernandez-Moran M., Fernandez-Vega I., Perez-Alvarez A., Villafani-Echazu J. (2016). Epstein–barr virus and multiple sclerosis. From evidence to therapeutic strategies. J. Neurol. Sci..

[117] Gallo A., Jang S.-I., Ong H.L., Perez P., Tandon M., Ambudkar I., Illei G., Alevizos I. (2016). Targeting the Ca^2+^ sensor STIM1 by exosomal transfer of EBV-miR-BART13-3p is associated with sjögren’s syndrome. EBioMedicine.

[118] Dasari V., Bhatt K.H., Smith C., Khanna R. (2017). Designing an effective vaccine to prevent epstein–barr virus-associated diseases: Challenges and opportunities. Expert Rev. Vac..

[119] Tensen C.P., Vermeer M.H. (2017). Microrna-155 potentiates tumour development in mycosis fungoides. Br. J. Dermatol..

[120] Cai L., Li J., Zhang X., Lu Y., Wang J., Lyu X., Chen Y., Liu J., Cai H., Wang Y. (2015). Gold nano-particles (AUNPS) carrying anti-EBV-miR-BART7-3p inhibit growth of EBV-positive nasopharyngeal carcinoma. Oncotarget.

